# From pharmaco-therapy to pharmaco-prevention: trends in prescribing to older adults in Ontario, Canada, 1997-2006

**DOI:** 10.1186/1471-2296-11-75

**Published:** 2010-10-07

**Authors:** Jana M Bajcar, Li Wang, Rahim Moineddin, Jason X Nie, C Shawn Tracy, Ross EG Upshur

**Affiliations:** 1 Undergraduate Medical Education, Faculty of Medicine, University of Toronto, Trillium Health Centre, CA Building 3rd Floor, 100 Queensway West, Mississauga, ON L5B 1B8 Canada; 2 Department of Pharmacy, Sunnybrook Health Sciences Centre, 2075 Bayview Ave., Room E3-05, Toronto, ON M4N 3M5 Canada; 3 Primary Care Research Unit, Sunnybrook Health Sciences Centre, 2075 Bayview Ave., Room E3-49, Toronto, ON M4N 3M5 Canada; 4 Leslie Dan Faculty of Pharmacy, University of Toronto, 144 College St., Toronto, ON M5S 3M2 Canada; 5 Department of Family and Community Medicine, University of Toronto, 263 McCaul St., 5th Floor, Toronto, ON M5T 1W7 Canada; 6 Institute for Clinical Evaluative Sciences, 2075 Bayview Ave., Room G1-06, Toronto, ON M4N 3M5 Canada; 7 School of Kinesiology and Health Science, York University, 344 Bethune College, Toronto, ON M3J 1P3 Canada; 8 University of Toronto Joint Centre for Bioethics, 155 College St., Suite 754, Toronto, ON M5T 1P8 Canada; 9 Dalla Lana School of Public Health, University of Toronto, 155 College St., 6th Floor, Toronto, ON M5T 3M7 Canada

## Abstract

**Background:**

The developed world is undergoing a demographic transition with greater numbers of older adults and higher rates of chronic disease. Most elder care is now provided by primary care physicians, who prescribe the majority of medications taken by these patients. Despite these significant trends, little is known about population-level prescribing patterns to primary care patients aged 65+.

**Methods:**

We conducted a population-based retrospective cohort study to examine 10-year prescribing trends among family physicians providing care to patients aged 65+ in Ontario, Canada.

**Results:**

Both crude number of prescription claims and prescription rates (i.e., claims per person) increased dramatically over the 10-year study period. The greatest change was in prescribing patterns for females aged 85+. Dramatic increases were observed in the prescribing of preventive medications, such as those to prevent osteoporosis (+2,347%) and lipid-lowering agents (+697%). And lastly, the number of unique classes of medications prescribed to older persons has increased, with the proportion of older patients prescribed more than 10 classes of medications almost tripling during the study period.

**Conclusions:**

Prescribing to older adults by family physicians increased substantially during the study period. This raises important concerns regarding quality of care, patient safety, and cost sustainability. It is evident that further research is urgently needed on the health outcomes (both beneficial and harmful) associated with these dramatic increases in prescribing rates.

## Background

The developed world is experiencing a demographic transition evidenced by population aging. Individuals are living longer and accumulating a greater burden of chronic diseases and, as a consequence, are utilizing healthcare services at greater rates [1,2]. These trends are expected to increase further as the "baby-boom" generation advances toward later life.

One of the standard interventions in chronic disease management is prescription medication. Medications were historically employed chiefly as treatments to alleviate symptoms; however, in the late 20^th ^and early 21^st ^centuries, medications have been increasingly utilized as preventive agents to modify and/or reduce health risks.

Most chronic disease management and medication prescription occurs in primary care by family physicians. Although data are available for overall medication use for all age groups [3] and for specific medical conditions, [4-6] little is known about population-level prescribing patterns for family physicians providing care to older adults. Therefore, we asked the following research questions:

1. What are the trends of medication prescription to individuals aged 65+ in the province of Ontario by family physicians?

2. What are the most commonly prescribed classes of medication, and have they changed over a 10-year interval?

3. Do prescription claims per person vary by patient age and sex?

## Methods

### Study cohort

We conducted a population-based retrospective cohort study to examine the prescribing trends of family physicians providing care to older adults over a 10-year period (January 1, 1997 to December 31, 2006). All Ontario residents aged 65+ who were eligible for universal public health insurance and who made at least one Ontario Drug Benefit (ODB) claim during the study period were included in the analysis. Patients without valid Ontario Health Insurance Plan (OHIP) numbers were excluded from the analysis.

### Data sources

Two administrative data sources were used to conduct the analysis. The ODB database includes data on all prescription medications dispensed to patients aged 65+ in Ontario, Canada. The Institute for Clinical Evaluative Sciences Physician Database (IPDB) is comprised of information from three sources: the Corporate Provider Database (CPDB), the Ontario Physician Human Resource Data Centre (OPHRDC) database, and the OHIP database of physician billings. The IPDB contains information about all licensed physicians in Ontario, including "physician specialty," which was used to extract family physicians for the present study. Physician specialty has two elements: functional and certified. While "certified" specialty derives from the physician's specialty certification, "functional" specialty is determined by the physician's activity-based specialty, which is assigned to each physician based on the combination of OHIP services provided, patient age and sex, and diagnoses that accounts for the highest proportion of the physician's total adjusted billings.

### Analysis

All individual medication names were sorted using existing medication sub-classes classification from the ODB database, after which medication sub-classes were grouped into specific medication classes. The medication classes were created to capture the potential indication for the use of the medications by inferring from the type of medications. Some medication classes were more specific than others. The 12 medication classes created through this process captured more than 80% of all medications prescribed for the present study period. All remaining classes - such as eye and ear preparations, cancer agents, and dermatological preparations - were collapsed into the "other classes" category.

Prescription claims were analyzed by patient age and sex. Population data from Statistics Canada were used in the calculation of age- and sex-specific rates. The degree of change for each group and the proportion that each group represents of the total was calculated for 2006 and compared to 1997. All statistical analyses were performed using SAS software version 9.1 (SAS Institute, Inc., Cary, NC). This study was approved by the Research Ethics Board at Sunnybrook Health Sciences Centre.

## Results

Over the 10-year period from 1997 to 2006, prescription claims increased dramatically for older adults in Ontario: from a total of 13,794,276 claims in 1997 to a total of 43,348,670 claims in 2006. This represents a 214% increase over the study period, which far exceeds the growth in the population of Ontario adults aged 65+ during this time period: from 1,384,739 in 1997 to 1,641,454 in 2006, an increase of 18.5%.

Table 1 shows the relative ranking of prescription classes in both 1997 and 2006 and the percentage change in rank order according to percentage increase over the study period. In both 1997 and 2006, cardiovascular medications were the most commonly prescribed medications, followed by psychotropic and gastrointestinal medications. All medication classes increased over the 10-year period, with several of the medication classes increasing by more than 200%. The lowest increases were for primarily symptom-based therapies (non-steroidal anti-inflammatory medications, antibiotics, asthma/chronic obstructive pulmonary disease therapies, and corticosteroids.) The steepest increases were for primarily preventive therapies: medications to prevent osteoporosis increased 2,347%, and lipid-lowering agents used to prevent cardiovascular disease increased 697%. Preventive medications and medications for chronic disease management have increased as a relative percentage of claims, while more primarily symptom-based medications such as analgesics, antibiotics, NSAIDS, COPD medications, and gastrointestinal medications have declined.

**Table 1:** Prescription claims by medication class for primary care patients aged 65+ in Ontario, Canada

MEDICATION CLASS	NUMBER OF CLAIMS			PROPORTION OF TOTAL CLAIMS			CLAIMS PER PERSON		
	**1997**	**2006**	**Change**	**1997**	**2006**	**Change**	**1997**	**2006**	**Change**

Osteoporosis	61,636	1,508,457	2,347%	0.38%	2.92%	668%	0.0445	0.919	1,965%
Lipid-lowering	445,390	3,549,066	697%	2.74%	6.88%	151%	0.3216	2.1621	572%
Thyroid replacements	484,499	1,724,859	256%	2.98%	3.34%	12%	0.3499	1.0508	200%
Psychotropics	1,899,995	6,716,502	254%	11.70%	13.02%	11%	1.3721	4.0918	198%
Cardiovascular	4,506,488	15,557,064	245%	27.75%	30.16%	9%	3.2544	9.4776	191%
Diabetes	686,009	2,341,826	241%	4.22%	4.54%	8%	0.4954	1.4267	188%
Gastrointestinal	1,764,359	5,326,708	202%	10.87%	10.33%	-5%	1.2741	3.2451	155%
Narcotics/analgesics	638,156	1,350,001	112%	3.93%	2.62%	-33%	0.4608	0.8224	78%
Corticosteroids	640,422	1,089,796	70%	3.94%	2.11%	-46%	0.4625	0.6639	44%
NSAIDs	1,094,162	1,816,538	66%	6.74%	3.52%	-48%	0.7902	1.1067	40%
Asthma/COPD	708,109	1,106,389	56%	4.36%	2.15%	-51%	0.5114	0.674	32%
Antibiotics	865,051	1,261,464	46%	5.33%	2.45%	-54%	0.6247	0.7685	23%
All other classes	2,619,665	7,685,790	193%	15.06%	15.96%	6%	1.8918	4.6823	148%

Also presented in Table 1 are the per person prescription claim rates for each of the unique classes of medication. (Compared to crude claims data, the use of 'claims per person' is advantageous as this measure controls for any change in the population of interest during the study period.) All classes of medication showed an increase in average annual claims per person. For example, for cardiovascular agents, in 1997 the average claims per person was 3.2544, whereas in 2006 the average claims per person was 9.4776, which represents an increase of 191%.

**Figure 1:**
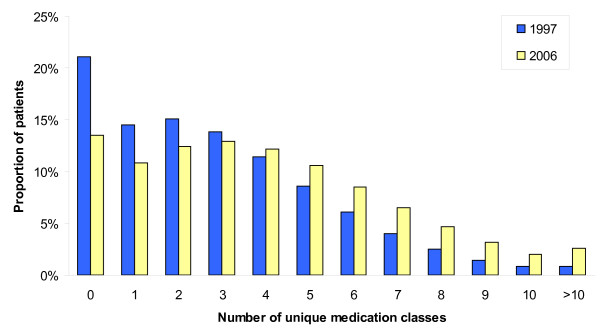
**Frequency distribution of number of unique medication classes for older adults aged 65+ in Ontario, Canada, 1997-2006**.

Figure 1 presents the distribution of the number of unique classes of medications prescribed to older adults in Ontario. Data is presented for the years 1997 and 2006. Over this 10-year period, the proportion of older adults making no prescription claims decreased by 36%, and the proportion of older adults taking one to three classes of medications decreased by 17%. Conversely, the proportion of older adults on four to nine classes of medication increased by 34%. Over the same time period, the proportion of older adults who were prescribed 10 or more classes of medication increased by 188%.

**Figure 2:**
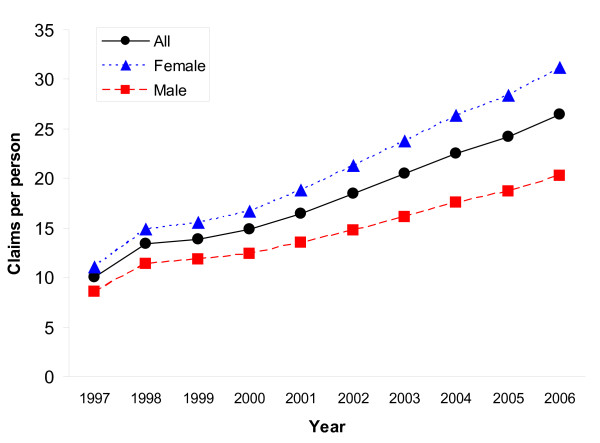
**Gender differences in prescription claims per person for older adults aged 65+ in Ontario, Canada, 1997-2006**.

Figure 2 presents the number of prescription claims per person by gender. As illustrated, claims per person were higher among females than males. In 1997, the overall prescription claims rate for Ontarians aged 65+ was 10 claims per person (female = 11; male = 8); by 2006, the overall claims rate increased to 26 claims per person (female = 31; male = 20).

**Figure 3:**
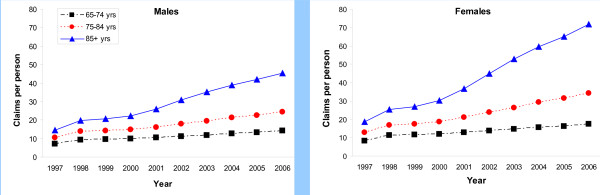
**Age differences in prescription claims per person for older adults aged 65+ in Ontario, Canada, 1997-2006**.

Figure 3 presents the number of prescription claims per person by age group for females and males, respectively. For the 10-year study period, there was an annual increase in the number of claims per person for both sexes. The rate of increase in the number of claims per person was greater for females than for males. This is true for all age groups: in the 65-74 age group, claims per person increased 107% for females and 101% for males; in the 75-84 age group, claims per person increased 170% for females and 132% for males; and, finally, in the 85+ age group, claims per person increased 286% for females and 210% for males.

## Discussion

The present study indicates dramatic increases in both absolute and per person prescription claims among primary care patients aged 65+ in Ontario, Canada over a 10-year time period. The year-over-year increases are greatest among older females, particularly those aged 85+. The data also demonstrate considerable increases in the number of classes of medications prescribed to older adults. Our data show the most significant increases in prescriptions occurring for risk factor management and chronic disease prevention and declines in relative terms of medications for symptom management. The data suggest a profound shift of clinical focus from treatment to preventive modalities in this population [7].

The increase in prescription claims cannot be explained entirely by increase in population, as the prescription claims per person is almost threefold higher than population increases. The prescription increases may be partially explained by greater reliance on clinical practice guidelines, [8,9] particularly more aggressive identification and management of risk factors for chronic conditions such as cardiovascular disease and osteoporosis. This trend towards increased use of preventive medications has resulted in a greater number of medication classes per patient, which has resulted in a shift in the profile of the typical elderly primary care patient. Whereas the regimen for a typical elderly primary care patient consisted of fewer than five classes of medications, now a greater proportion of these patients are on more than five classes. Taken together, these findings raise important questions concerning quality of care, safety, and cost.

In terms of quality of care, it is difficult to discern the benefits associated with the steep increase in prescriptions, particularly the increase in the number of classes of medications prescribed and the steep increases in the oldest age groups (i.e., 85+). There is increased use of clinical guidelines to guide medication prescribing; however, there are few clinical trials that have included elderly patients and so all claims for the effectiveness of such therapies with elderly patients are extrapolations from trials of younger patients [10]. As well, the rising rates of prescribing speak to the increasing complexity of clinical care. Increasing medication rates might explain, at least in part, the observed increase in visits to their family physician [1]. Increased medication prescribing creates complex treatment regimens. For physicians, this added complexity results in the need for more intense monitoring and increased management of side effects. For patients and their families, following complex medication regimens is difficult and burdensome; therefore, adherence rates are often low [11,12]. Taken together, then, the complexity involved with complex medication regimens results in cases that cannot be properly managed in conventional primary care visits [13].

The dramatic increase in prescriptions also raises concerns for patient safety and the potential for iatrogenic harm, particularly as many older adults now take multiple concomitant medications [14-16]. A number of recent studies have raised potential concerns with increased use of medications such as antipsychotic medications in the older and vulnerable adults [17]. The interactions of many of these medications are unknown, but recent research has shown the possibility of many harmful interactions, especially in older adults who have physiologic changes that impact medication clearance and pharmaco-dynamics [18]. There is a risk that increasing medication use, even for appropriate indications, may initiate a "prescribing cascade" where a medication causes an adverse effect for which another medication then is prescribed, which then in turn can cause another adverse effect for which another medication is prescribed [19]. Therefore, it is imperative that future studies evaluate the harms and benefits of complex medication regimens.

The increases in prescription claims per person documented in this study provide the basis for further exploration of associated cost implications. With the baby boom generation about to enter retirement age, publicly-funded drug programs must assess their ability to bear the costs associated with dramatic increases in prescribing rates. A recent report published by the Canadian Institutes of Health Information (CIHI) noted that the top 10 medications prescribed for older adults in six Canadian provinces account for 48% of all medication expenditures on older adults [20]. It is important to note, however, that this report did not include data from Ontario, British Columbia, or Quebec as prescription claims from these provinces are not submitted to the National Prescription Drug Utilization Information System database that was used for this report. Similar increases in use of prescription medications in the past decade have been observed in England. In 1997, adults aged 60+ were prescribed 22.3 medications per year on average; by 2006, this had increased to 42.4 medications per year [21]. There is also evidence demonstrating that nearly half of the total expenditures on prescription medications are accounted for by 5% of the population; nearly half of these high-cost users were over the age of 65 [3].

The greatest strength of this study is its population base. We were able to capture all prescription claims generated by family physicians for all eligible older Ontarians in the study period. As noted above, the recent CIHI report did not include data from Ontario, which is Canada's most populous province. A limitation is that we do not have data for over-the-counter medications, which can be numerous for older adults, [22,23] so our data underestimate overall medication usage. Our data do not reflect actual medication consumption by patients, nor do our data reflect the actual number of prescriptions that were written, only those that were brought to the pharmacy and dispensed. Since poor adherence of medication regimens is common, including not bringing a prescription to a pharmacy, the methodology used in this study does not capture the actual medications prescribed nor the actual amount consumed.

## Conclusions

These data indicate dramatic increases in prescription claims in the province of Ontario, Canada over a recent 10-year time span. These increases, particularly among older females, cannot be explained by population increase and are unlikely the result of increased disease burden. The observed increases in prescribing rates, especially in the number of unique classes of medications, have important implications for quality of care, safety, and cost [24]. Given the growing concern regarding polypharmacy, given the increasingly-complex medication regimens prescribed to older patients, and given the absence of detailed knowledge of possible medication interactions, it is evident that further research is urgently needed on the health outcomes (both beneficial and harmful) associated with these dramatic increases in prescribing rates.

## Competing interests

The authors declare that they have no competing interests.

## Authors' contributions

JMB contributed to the design of the study, participated in the data analysis, drafted the first version of the manuscript, and contributed to subsequent revisions. LW contributed to the design of the study, performed the data analysis, and contributed to the drafting and revising of the manuscript. RM contributed to the design of the study, participated in the data analysis, and contributed to the drafting and revising of the manuscript. JXN contributed to the design of the study, participated in the data analysis, and contributed to the drafting and revising of the manuscript. CST contributed to the design of the study, participated in the data analysis, and contributed to the drafting and revising of the manuscript. REGU conceived and designed the study, participated in the data analysis, contributed to the drafting and revising of the manuscript, and will act as study guarantor. All authors have read and approved the final version of the manuscript.

## Pre-publication history

The pre-publication history for this paper can be accessed here:

http://www.biomedcentral.com/1471-2296/11/75/prepub
